# Total Pulpotomy Using a Newer Mineral Trioxide Aggregate for Managing Complicated Crown Fractures in Mature Permanent Incisors: A Case Report

**DOI:** 10.7759/cureus.68252

**Published:** 2024-08-30

**Authors:** Anurag Negi, Sakshi Katyal, Pranitha Vallala, Hima K Bindu, Vijay Yadav, Shamimul Hasan

**Affiliations:** 1 Department of Dentistry, All India Institute of Medical Sciences, Bibinagar, IND; 2 Oral Medicine and Radiology, Faculty of Dentistry, Jamia Millia Islamia, New Delhi, IND

**Keywords:** mineral trioxide aggregrate, bioceramic materials, full pulpotomy, tooth fracture, dental trauma

## Abstract

Crown fractures with pulp exposure in mature permanent teeth present a challenging situation that requires immediate attention. Mineral trioxide aggregate (MTA) as a sealing material after pulpotomy has proven to be a reliable treatment in these cases compared to traditional root canal therapy. This case report emphasizes the importance of early diagnosis and careful treatment planning for complicated crown fractures and concussion injuries in a mature permanent incisor of a young child. Total pulpotomy using MTA has proven to be an effective treatment for fractures in young mature incisors with pulp exposure, as evidenced by a one-year follow-up in our case, which showed no discoloration.

## Introduction

Traumatic injuries to teeth and supporting structures are an emergency condition primarily seen in young individuals aged six to 12 years who experience falls or accidents [1,2]. School children with young permanent teeth often sustain such injuries (25%), with class II division 1 being the most common type of dentition. The most frequently affected teeth are the maxillary central and lateral incisors [3]. Dental fractures are classified according to the fractured tissue and pulp involvement and include enamel infractions, uncomplicated crown fractures (enamel fractures and enamel-dentin fractures), complicated crown fractures (enamel-dentin fractures with pulp exposure), and crown-root and root fractures [4]. Complicated crown fractures (those with pulp exposure) in permanent teeth may constitute one-third of all traumatic dental injuries [1-3]. Luxation injuries are more common in primary teeth, whereas crown fractures are more frequent in permanent teeth due to the more rigid bony support in permanent dentition [5]. Concurrent injuries of different types in the same teeth can adversely impact the prognosis. Such cases require detailed clinical and radiographic evaluation to ensure accurate diagnosis, timely intervention, and the prevention of potential late complications [6].

Early treatment of crown fractures with pulp exposure seeks to maintain pulp vitality, thereby supporting continued root development, apex closure, and thickening of the root dentin [7]. Failure to comply with this recommendation can result in further treatment difficulties, particularly in immature permanent teeth with thin and wide roots, open apices, and delicate dentinal walls. Studies indicate that immature teeth are more vulnerable to root fractures than mature teeth because of their thin and weak root dentin. Therefore, maintaining pulp vitality in mature permanent teeth of individuals aged 11-12 years is recommended. This strategy encourages the thickening of root dentin by continuously depositing secondary and tertiary dentin in the cervical region, thus helping to lower the risk of future root fractures [7,8].

In the case of complicated crown fractures, vital pulp therapy (VPT) interventions include direct pulp capping (DPC), partial pulpotomy (PP), and complete pulpotomy (CP). In DPC, a protective material is placed directly over the exposed pulp area. This procedure is used for recently exposed vital pulp with a pinpoint-sized exposure. PP involves the partial removal of the coronal pulp, followed by hemostasis and the application of a pulp-capping material. It is performed in cases of pulp exposure that are managed within 14 days of trauma, without caries, with an open apex or thin dentinal walls, and vital, asymptomatic pulp. CP involves the complete removal of the coronal pulp down to the canal orifices, followed by hemostasis and the application of a pulp-capping material. This is typically done when there is a delay of more than two weeks between the trauma and treatment, resulting in extensive pulp exposure [9]. These fractures can greatly impact an individual's emotional, psychological, and social well-being and also hamper their oral health-related quality of life (OHRQoL) [10].

Recently, partial or full pulpotomy has gained significant research interest as a definitive treatment for mature permanent teeth, owing to advancements in bioactive materials such as MTA and improved insights into pulp biology. However, a major drawback of using MTA in incisors is its potential to cause discoloration [11]. A novel Biostructure MTA (Safe Endo, India) has been introduced to address the discoloration issue associated with bismuth oxide by substituting it with zirconium dioxide [12].

Partial or Cvek pulpotomy entails the removal of inflamed coronal pulp while maintaining the deeper, cell-rich pulp, which is presumed to be unaffected by inflammation [13]. Cvek et al. reported that after a complicated crown fracture, hemorrhage and injury at the odontoblastic level did not surpass 2 mm on the pulp exposure side within three hours [14]. After 48 hours, it extended from 1.5 to 2 mm, and after seven days, it extended from 0.8 to 2.2 mm. In pulps exposed for 48 hours to seven days, nearly 36% showed proliferated pulp reactions [15]. Other studies also reported that up to 48 hours after traumatic injury, inflammation is limited to 2 mm pulpal depth as the initial reaction of the pulp is proliferative. As time passes more than 48 hours, chances of direct bacterial contamination of the pulp increase with the zone of inflammation progressing apically. Studies also suggest that a delay of up to nine days between damage onset and treatment may have minimum consequences in the outcome of Cvek’s pulpotomies [16,17]. However, traumatic exposures after 72 hours and a carious exposure with symptoms of irreversible pulpitis are two examples in which this total pulpotomy may be indicated [8]. The severity of the initial trauma, the extent of pulp exposure, any associated luxation injuries, and the time elapsed between the incident and medical treatment can all significantly affect the pulp's recovery [18].

Endodontic treatment (RCT) is a practical, albeit conventional, alternative to VPT for mature permanent teeth that have crown fractures with pulp exposure [19]. Revitalization and apexification are the preferred treatments for necrotic immature permanent teeth. While these approaches effectively address the disease, they may leave the root walls thin and vulnerable to fracture due to unpredictable root development. The success of orthodontic intervention in such cases remains unclear, given the limited evidence on the feasibility of orthodontic movement following revitalization procedures [20]. Therefore, in cases of traumatic pulp exposure in young patients, the key objective is to preserve pulp vitality, even if apex closure is not accomplished [20,21].

Mineral trioxide aggregate (MTA) is a tricalcium silicate-based material that has successfully replaced calcium hydroxide (CH)-based cement as a pulp-capping agent in pulpotomy procedures [22]. MTA's effectiveness as a pulpotomy agent is due to its favorable properties, such as excellent biocompatibility, bioactivity, alkaline pH, and the formation of denser, more uniform dentin bridges compared to calcium hydroxide. The lack of tunnel defects in the dentin bridges formed by MTA enhances its ability to seal against microbial infiltration, which may contribute to improved clinical outcomes [23,24].

This clinical report aims to describe the early conservative management of traumatized mature permanent maxillary incisor teeth with pulp exposure using a novel MTA formulation.

## Case presentation

A young male, aged 11.5 years, visited our outpatient department with a complaint of pain in his fractured left maxillary incisors. The injury occurred seven days ago during a fall while playing football, and he was very concerned about his appearance.

**Figure 1:**
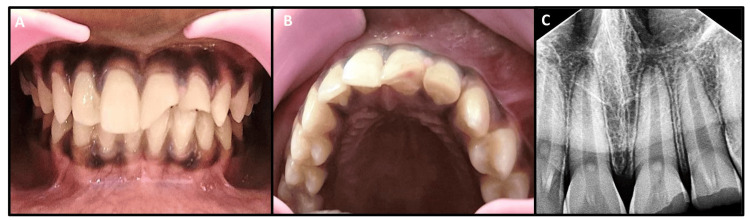
Preoperative clinical and radiographic pictures. Three periodical X-rays were taken at 60, 90, and 110 degrees to rule out root fractures. However, a 90-degree X-ray was taken for standardization. Intra-oral front view (A) and occlusal view (B) show an enamel-dentin fracture with pinpoint pulp exposure in the left central incisor (#21), an enamel-dentin fracture without pulp involvement in the left lateral incisor (#22), and an intact right central incisor (#11). Preoperative intra-oral radiograph depicts periodontal ligament (PDL) widening in teeth #11 and #21 (C).

A thorough clinical examination showed an enamel-dentin fracture with pinpoint pulp exposure in the left central incisor (#21) and an enamel-dentin fracture without pulp involvement in the left lateral incisor (#22). The right central incisor (#11) was intact. None of the teeth exhibited spontaneous pain. Cold tests (Roeko, Endo-Frost, Coltene/Whaledent, Germany) and electric pulp tests (EPT; Waldent, WL23HG107) were positive in #11, #21, and #22. An early response with lingering pain for two minutes was seen in #21 (at lower current on EPT) when compared to adjacent and contralateral teeth, and #12, #13, and #23 were taken as control. Baseline EPT values were recorded for #11, #21, and #22 (#11: current 25, #21: current 10, and #22: current 22). Pain from the cold test persisted for 10 minutes in #21, while the pain in #22 subsided within seconds after the stimulus was removed. No pain was detected upon palpation. The percussion test revealed slight tenderness in #11 and #21. The transient apical breakdown seen in concussion or subluxation injuries was observed in #11 and #21, while the peri-radicular area of tooth #22 appeared normal. No root fractures were found in #11, #21, or #22 (Figure 1, Panels A-C).

A diagnosis of concussion for #11, concussion with an Ellis class III fracture and irreversible pulpitis for #21, and an Ellis class II fracture with reversible pulpitis for #22 was established based on the classification of crown fracture and luxation injuries given by International Association of Dental Traumatology (IADT) and American Academy of Pediatric Dentistry (AAPD). The treatment plan based on IADT and AAPD guidelines included pulpotomy with composite restoration for #21, composite restoration with a calcium hydroxide liner for #22, and a wait-and-watch approach for #11. The patient and the parents were informed about the plan and reassured regarding aesthetic outcomes. Written consent was obtained from the patient’s parent for the treatment and publication, after an explanation in both Telugu and English.

During the first appointment, 1.8 ml of lidocaine HCL 2% with 1:80,000 epinephrine was administered via local infiltration to anesthetize #21 (Lox 2%, Neon, Maharashtra Industrial Development Corporation (MIDC), Tarapur, Maharashtra, India). Under rubber dam isolation, #22 was restored with a calcium hydroxide liner (Dycal, LD Caulk Co./Dentsply, Milford, Delaware) applied to the center of the dentinal surface and covered with a direct composite restoration (Nanofilled composite, Filtek Z350XT, 3M, India). A selective etch technique was employed, where only the enamel was etched after beveling the facial surface and rinsed. Calcium hydroxide was then applied to the center of the dentin, which was covered by a resin-modified glass ionomer cement (RMGIC) liner. A universal bonding agent was applied to both the enamel and dentin and cured. This was followed by a freehand composite restoration.

An access opening was then made for #21 using a round and straight bur. A sterile diamond round bur was used to remove the pulp, rotating at high speed with an air-rotor handpiece and ample water irrigation. The entire coronal pulp was removed to achieve a 2-3 mm chamber depth for the insertion of sealing material. The pulp chamber was irrigated alternately with 3% sodium hypochlorite and normal saline, which controlled bleeding within three to four minutes. Literature has reported the use of 0.5%-5% NaOCl use, as it acts as an antibacterial and dissolves inflamed pulp [25]. Hemostasis should be ideally achieved in approximately five minutes by applying a cotton/gauze pellet soaked with 2.5% NaOCl. Failure to achieve hemostasis after pulpal bleeding indicates pulpal tissue that is irreversibly inflamed. The operator should proceed to the next level of the treatment plan [26].

MTA (Biostructure MTA, SafeEndo, India) powder and liquid were mixed to a paste-like consistency with a plastic spatula and applied over the radicular pulp with a hand condenser in a thickness of 2 mm. A cotton pellet soaked in distilled water was placed over the MTA and covered with a temporary restoration (Cavit™G, 3M ESPE, Germany). There were two reasons to do the procedure in two visits: Previous recommendations suggested a two-visit MTA pulpotomy, where wet cotton was placed over the MTA to facilitate hydration, leading to a proper setting with minimal expansion and a better seal. Given that the time between injury and treatment exceeds 72 hours, a follow-up of 24 hours after completing the MTA pulpotomy was necessary. This follow-up ensured that if symptoms of irreversible pulpitis persisted, reentry into the pulp chamber could be more easily performed to proceed with a pulpectomy.

**Figure 2:**
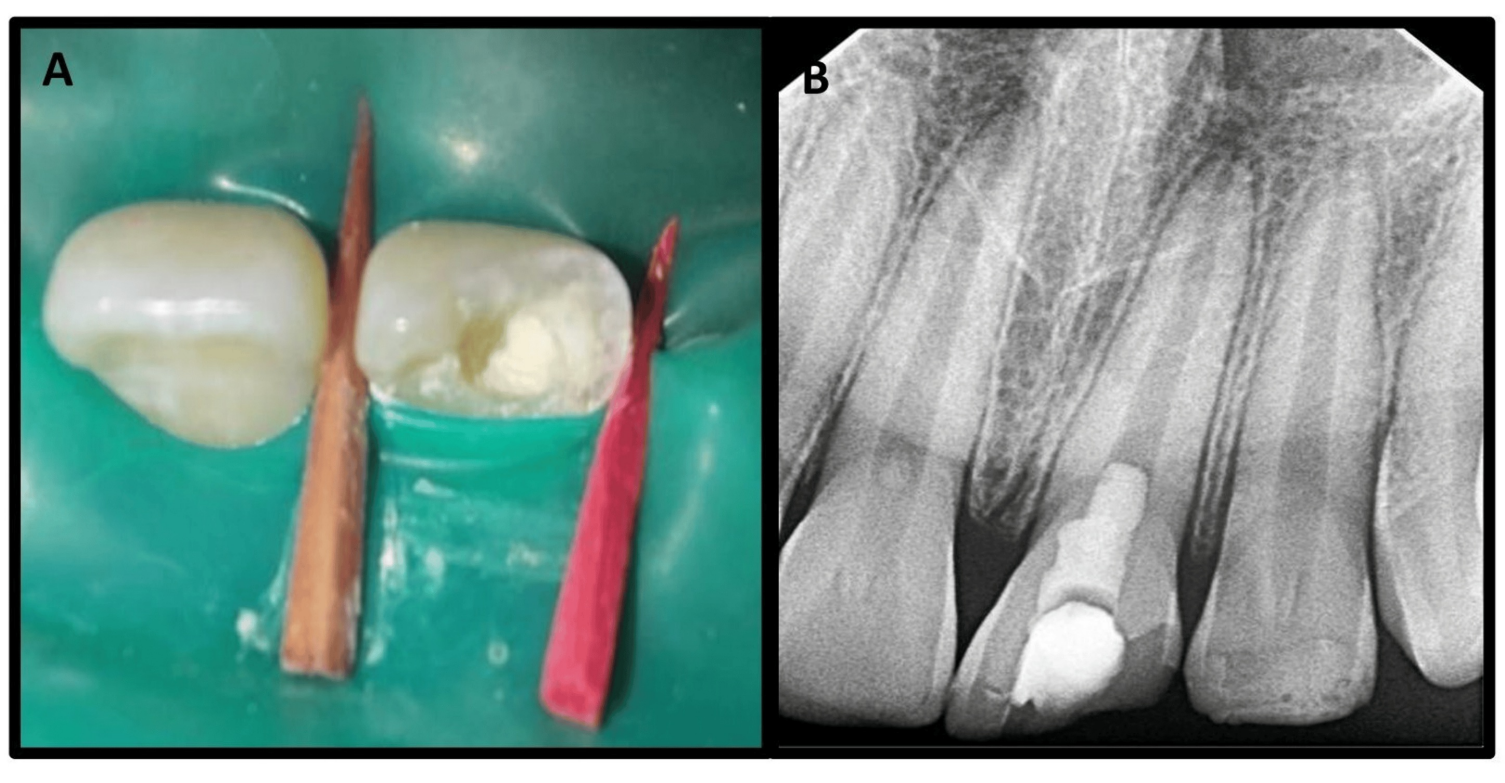
Post-treatment clinical and radiographic pictures Intra-oral picture showing mineral trioxide (MTA) placement after pulpotomy in #21 (A). Post-treatment intra-oral radiograph after placement of MTA covered with wet cotton and temporary restoration in #21 and composite restoration in #22 (B).

A post-procedure radiograph revealed MTA covered with wet cotton and a temporary restoration in #21 as well as a composite restoration in #22 (Figure 2, Panels A and B).

**Figure 3:**
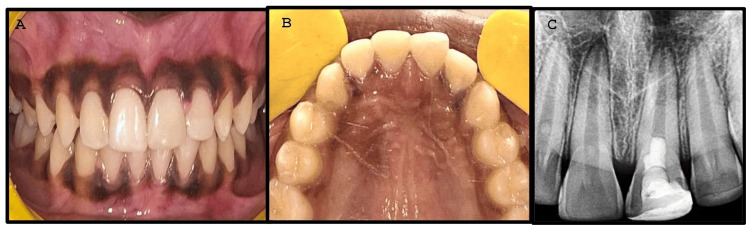
Post-treatment clinical and radiographic pictures (after 24 hours) Intra-oral view (A) and occlusal view (B) of polished restoration on #21 and #22. Intra-oral radiograph showing mineral trioxide (MTA) covered with glass ionomer cement and composite restoration (C).

The next appointment was scheduled 24 hours later. Reentry into tooth #21 was carried out, the cotton was removed, and a 2-mm thick glass ionomer restoration was applied directly over the MTA. Shade matching was done, followed by selective etching and direct composite restoration in #21. The restoration was then finished and polished using SuperSnap (Shofu, Japan). A post-treatment radiograph revealed MTA covered with glass ionomer cement and composite restoration (Figure 3, Panels A-C).

The patient was scheduled for a follow-up visit one month later. At the follow-up visit, the patient was asymptomatic upon clinical examination. All involved teeth responded positively to cold testing and EPT, although the EPT response in #21 required a higher current. Delayed response at the current of 55 was seen for #21 due to the presence of MTA material in the pulp chamber after CP. This is a positive sign indicating vital pulp tissue in the radicular portion. There was no tenderness to percussion, no pain on palpation, grade I mobility, and no periodontal pockets.

**Figure 4:**
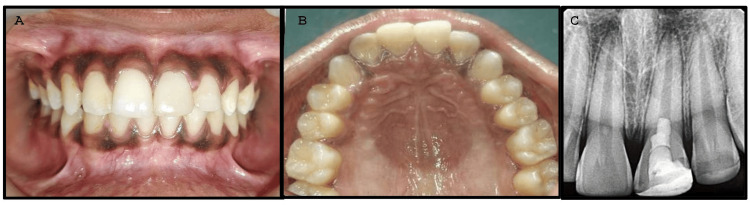
Clinical and radiographic pictures after one year of follow-up

The patient was evaluated at three months, six months, and one year. Clinical and radiographic assessments were conducted at each follow-up visit. The patient continued to be asymptomatic, and the radiographs showed no changes, with a normal peri-radicular area after one year of follow-up (Figure 4, Panels A-C).

## Discussion

Guidelines issued by international societies aid dentists in clinical decision-making and delivering appropriate patient care. The AAPD, IADT, and European Society of Endodontology (ESE) currently provide position statements and guidelines for managing dentoalveolar traumatic injuries [6,20,21]. According to the IADT guidelines, a wait-and-watch approach with regular follow-up is recommended for concussion injuries [6]. In our case, these guidelines were used as a reference for managing crown fractures and luxation injuries. The maxillary right incisor was diagnosed as a concussed tooth. Baseline EPT score was recorded for future comparison of vitality response as the tooth may respond negatively to EPT and cold test for two to three weeks after trauma but that does not indicate loss of vitality of the tooth. Baseline EPT was recorded, and the tooth's tenderness subsided without any intervention within four weeks.

The AAPD and IADT advise that crown fractures with pulp exposure in both mature and immature permanent teeth should be managed with VPT, including pulp capping or pulpotomy [6,20]. However, research has shown that pulpotomy has a higher success rate than DPC in mature permanent teeth. This approach aims to preserve the vitality of young radicular pulp, which supports continued root thickening and increased fracture resistance. Studies indicate that treating a complicated crown fracture within 48 hours significantly improves the chances of pulpal recovery [14,20]. While the time elapsed after an injury is important, it should not be the only factor in determining the treatment plan. PP is the preferred treatment if performed within 24-48 hours. If more than 72 hours have passed, a CP or pulpectomy may be necessary. In this case, the trauma was reported after one week, but clinical examination revealed only symptomatic irreversible pulpitis. The patient was 11.5 years old, near the borderline for root apex maturity. Consequently, we opted for a CP using a newer bioceramic material.

The success of calcium hydroxide pulpotomy is influenced by both the size of the pulpal exposure (<4 mm) and the interval between the injury and treatment [13,14]. Studies have indicated that inflammation may be restricted to the coronal area up to 2 mm even seven days after the injury [10,14]. AAPD accepts MTA as a pulpotomy capping agent [20]. Given this, a total pulpotomy with MTA capping was performed to manage the complicated crown fracture of the left maxillary incisor, followed by direct composite restoration the following day.

The AAPD and IADT recommend sealing dentinal tubules as soon as possible in cases of uncomplicated crown fractures involving enamel and dentin. The selective etch technique provides better bonding to enamel and dentin than the total-etch technique [6,20]. Accordingly, in this case, the dentinal tubules of the maxillary left lateral incisor were sealed at the initial appointment with a calcium hydroxide liner, followed by immediate permanent restoration using direct composite with the selective etch technique. The selective etch technique was used, starting with etching and rinsing the enamel after beveling the facial surface. Both enamel and dentin were then covered with a universal bonding agent before applying a composite restoration. After etching the enamel, calcium hydroxide was applied and covered with a light-cured RMGIC.

In VPT, younger pulp with an open apex typically has a better prognosis than older pulp, which is often more fibrotic and less capable of healing. As a result, older patients may face less favorable outcomes with vital pulp therapy [16,27].

In this clinical case, the patient was a young male with a closed apex who had developed roots in teeth #11 and #21. However, previous research has challenged this view, suggesting that the success of Cvek pulpotomies is not influenced by the apical status (whether open or closed) at the time of treatment [14]. Pulp removal was performed near the cementoenamel junction to eliminate all inflamed pulp tissue in the coronal region, resulting in a prepared space capable of accommodating the 2-3 mm MTA. This approach is consistent with research findings, which suggest that bleeding and damage at the odontoblastic level do not extend beyond 2.2 mm even after seven days of trauma, and only 36% of exposed pulps exhibit proliferated pulp responses within 48 hours to seven days [14].

A sterile round bur, used with a high-speed handpiece and water coolant, was employed to excise the pulp, providing a sharp and precise incision compared to a spoon excavator. Hemostasis was achieved using 3% hypochlorite within three to four minutes, which is a positive indicator of inflammation-free radicular pulp. Research indicates that achieving hemostasis within 6-10 minutes after pulpotomy enhances the likelihood of pulp survival [20,21].

MTA was selected as the pulp-capping agent in this case due to its acceptance and recommendation by the AAPD. Evidence confirms its effectiveness as the most successful pulp-capping material [20]. Studies have indicated that MTA demonstrates either equivalent or superior performance as a direct pulp-capping agent than calcium hydroxide, even over extended periods of observation, despite drawbacks such as increased cost and tooth discoloration associated with MTA [28,29]. The discoloration mainly results from the oxidation of heavy metal oxides, such as bismuth or iron, and the interaction between erythrocytes and unset cement when hemostasis is inadequate. To prevent this, several strategies have been suggested, including using cement with alternative radiopacifying agents like zirconium oxide, sealing the surrounding dentinal tubules with dentin-bonding agents before applying MTA, or adding zinc oxide or aluminum fluoride to the powder [9].

However, with the latest MTA formulations, discoloration is minimal, as most of the discoloration is due to the MTA itself rather than infiltrated dentin [30]. In this case, Biostructure MTA, a novel formulation, was used for pulp capping, and no discoloration was observed in tooth #21 even after a one-year follow-up. This lack of discoloration may be attributed to the new MTA composition, which uses zirconium oxide as the radiopaque agent instead of the bismuth oxide used in previous formulations as well as the placement of MTA at the cementoenamel junction. Additional benefits of Biostructure MTA include its ability to harden in the presence of moisture and blood, controlled setting time, high mechanical resistance, excellent biocompatibility, low solubility, high alkalinity, and microbicidal activity [12]. Hence, it is strongly recommended to choose full pulpotomy with Biostructure MTA rather than PP for treating crown fractures with pulp exposure in mature permanent teeth.

For #22, a 0.5-mm thickness of dentin over pulp was seen on the radiograph, so a liner of calcium hydroxide was applied and covered with direct composite per the literature [6,20,21]. The immediate final restoration of the pulpotomized tooth was done with composite restoration to provide dentinal tubule sealing and allow reparative dentin formation over the fractured site. Immediate restoration resulted in a regain of emotional and social confidence in this young child.

In our case, the three affected teeth exhibited different types of traumatic injuries, each requiring distinct management strategies. A comprehensive treatment plan tailored to the patient's needs was developed. Prompt management of the fractured teeth and aesthetic direct composite restoration helped restore the patient's self-esteem and prevent long-term psychosocial trauma. A similar case report detailing successful management of a combination of dental injuries from trauma is documented in the literature [31].

## Conclusions

This case report provides a guide for comprehensive diagnosis and treatment planning for traumatic dental injuries in young children with mature permanent teeth. Managing dental traumatic injuries can be quite challenging, especially in complex cases involving multiple levels of injury. Accurate diagnosis and effective treatment planning are crucial for achieving successful outcomes. Pulpotomy using novel MTA is a dependable technique for promoting the thickening of root dentin in young mature teeth with crown fractures and pulp exposure. Early restoration with direct composite will enhance aesthetics and boost the young child's self-confidence.
